# Effects of sub-chronic amylin receptor activation on alcohol-induced locomotor stimulation and monoamine levels in mice

**DOI:** 10.1007/s00213-020-05607-8

**Published:** 2020-07-10

**Authors:** Aimilia Lydia Kalafateli, Cajsa Aranäs, Elisabet Jerlhag

**Affiliations:** Institute of Neuroscience and Physiology, Department of Pharmacology, The Sahlgrenska Academy at the University of Gothenburg, POB 431, SE-405 30 Gothenburg, Sweden

**Keywords:** Amylinergic pathway, IAPP, Mesolimbic dopamine system, Reward, Alcohol use disorder, Calcitonin, Calcitonin receptor, Neurotransmitters

## Abstract

**Rationale:**

Amylin receptors consist of the calcitonin receptor (CTR) and one of three receptor activity-modifying proteins (RAMPs). The identification of amylin receptors in areas processing reward, namely laterodorsal tegmental area (LDTg), ventral tegmental area (VTA), and nucleus accumbens (NAc), has attributed them a role as reward regulators. Indeed, acute activation of amylin receptors by the amylin receptor agonist salmon calcitonin (sCT) attenuates alcohol-induced behaviours in rodents.

**Objectives:**

The effects of long-term administration of sCT on alcohol-related behaviours and the molecular mechanisms underlying these processes are not yet elucidated. To fill this knowledge gap, we investigated the effects of sub-chronic sCT treatment on the locomotor stimulatory responses to alcohol in mice and the molecular pathways involved.

**Methods:**

We assessed the behavioural effects of sub-chronic sCT treatment by means of locomotor activity experiments in mice. We used western blot to identify changes of the CTR levels and ex vivo biochemical analysis to detect changes in monoamines and their metabolites.

**Results:**

After discontinuation for 5 days of sCT treatment, alcohol did not induce locomotor stimulation in mice pre-treated with sCT when compared with vehicle, without altering secondary behavioural parameters of the locomotor activity experiment or the protein levels of the CTR in reward-related areas in the same set of animals. Moreover, repeated sCT treatment altered monoaminergic neurotransmission in various brain areas, including increased serotonin and decreased dopamine turnover in the VTA. Lastly, we identified a differential effect of repeated sCT and acute alcohol administration on alcohol-induced locomotion in mice, where sCT initially attenuated and later increased this alcohol response. It was further found that this treatment combination did not affect secondary behavioural parameters measured in this locomotor activity experiments.

**Conclusions:**

These data suggest that sub-chronic sCT treatment differentially alters the ability of alcohol to cause locomotor stimulation, possibly through molecular mechanisms involving various neurotransmitter systems and not the CTR levels per se.

**Electronic supplementary material:**

The online version of this article (10.1007/s00213-020-05607-8) contains supplementary material, which is available to authorized users.

## Introduction

The pancreatic hormone amylin is physiologically involved in the inhibition of insulin secretion, gastric emptying, and glucagon secretion (Hay et al. 2015). The anoregixenic properties of endogenous amylin and its receptor agonist, salmon calcitonin (sCT), are well established (Lutz et al. 1995; Whiting et al. 2017; Wielinga et al. 2010). To reduce food intake, both amylin and sCT act on central amylin receptors, which consist of a core calcitonin receptor (CTR) and one of the three receptor activity-modifying proteins (RAMP) 1, 2, or 3, (Braegger et al. 2014; Potes et al. 2010; Rushing et al. 2001). Amylin receptors in reward-related areas such as the laterodorsal tegmental area (LDTg), the ventral tegmental area (VTA), and nucleus accumbens (NAc) regulate food intake (Baisley and Baldo 2014; Mietlicki-Baase et al. 2015a; Reiner et al. 2017). In addition to food intake, those receptors seem to mediate the effect of sCT to reduce energy balance (Mietlicki-Baase and Hayes 2014; Mietlicki-Baase et al. 2017; Mietlicki-Baase et al. 2013).

Recent data suggest that activation of amylin receptors by sCT attenuates alcohol-mediated behaviours in rodents (Kalafateli et al. 2019a; Kalafateli et al. 2019b) and that the gene expression of amylin receptor components in the NAc is differential in low compared with high alcohol-consuming rats (Kalafateli et al. 2019a). In particular, acute sCT decreases alcohol-induced locomotor stimulation, accumbal dopamine release, and alcohol-induced CPP in mice. Moreover, it reduces alcohol intake, blocks the alcohol deprivation effect, and decreases lever responses for alcohol reward in rats. Those studies have attributed novel properties to sCT as a modulator of alcohol-mediated behaviours, making it an interesting candidate for further alcohol research. Albeit these initial studies showing interaction of the amylinergic pathway with alcohol-mediated behaviours, the effects of sub-chronic sCT administration on alcohol-induced locomotor stimulation in mice and the identification of some underlying brain molecular pathways remain unknown. Therefore, we examined the effects of an acute alcohol challenge on locomotor activity in mice, following termination for 5 days of daily sCT pre-administration. Furthermore, we performed western blot experiments to identify whether the above behavioural results are linked to changes in the protein levels of either CTR isoform “a” or “b” in the LDTg, VTA, and NAc of the same mice. In a further attempt to explain the behavioural outcomes of the repeated sCT locomotor activity experiments, we examined the effects of 5 days of repeated sCT administration on the levels of monoamines and their metabolites in various brain reward-related areas, including the VTA, NAc, hippocampus, dorsal striatum, prefrontal cortex (PFC), and amygdala, in a separate set of mice. Lastly, we evaluated the effects of 5 days of repeated sCT and alcohol administration on the daily locomotor activity in mice.

## Materials and methods

### Animals

For the present experiments, adult post-pubertal age-matched male NMRI mice (8–12 weeks old and 25–30-g body weight; Charles River, Susfeldt, Germany) were used since they display robust locomotor stimulation as a response to alcohol (Jerlhag et al. 2009). The mice were group housed, fed ad libitum, and maintained at a 12/12 h light/dark cycle and at 20 °C with 50% humidity. An independent set of mice was used in each experiment, with the exception of the western blot data, where the brains from the first behavioural test were used. All experiments were approved by the Swedish Ethical Committee on Animal Research in Gothenburg.

### Drugs

sCT (Tocris Bioscience, Bristol, UK) was diluted in vehicle (0.9% sodium chloride solution) and administered intraperitoneally (IP) at the dose of 5 μg/kg. The agonist was administered 30 min prior to alcohol administration in the experimental setup, where alcohol was administered the same day with sCT. This dose of sCT was used, as we have previously established that this dose attenuates alcohol-mediated behaviours in rodents (Kalafateli et al. 2019a; Kalafateli et al. 2019b). Moreover, this sCT dose, at least acutely, does not affect blood alcohol concentration (Kalafateli et al. 2019b), rendering the possibility of pharmacokinetic interaction between the two drugs less likely. Alcohol (95%, Solveco AB; Stockholm, Sweden) was diluted in vehicle (0.9% sodium chloride solution) to 15% *v*/v and was administered at a dose of 1.75 g/kg, IP, for all experiments. This alcohol dose is established to cause locomotor stimulation and increased dopamine release in mice (Jerlhag et al. 2009). All drugs were diluted in such a way that each animal received the volume of 1/100 of their body weight in ml.

### Locomotor activity

Locomotor activity was registered in six sound-attenuated, ventilated, and dimly lit locomotor boxes (420 × 420 × 200 mm; Open Field Activity System, Med Associates Inc., Georgia, Vermont, USA). In this system, 15 × 15 infrared beams at the bottom of the floor allow a computer-based system to register the locomotor activity of each mouse per 5 min during the entire time defined by the protocol.

In all locomotor activity experiments, the main analysed parameter was ambulatory distance, as drugs of abuse stimulate locomotion and increase the distance travelled in the boxes (Liljequist et al. 1981; Zombeck et al. 2010). This can be considered as an indirect measurement of alcohol’s ability to activate the mesolimbic dopamine system (Phillips and Shen 1996). To further investigate whether treatment affects secondary behavioural parameters of this experiment, we additionally analysed ambulatory counts, ambulatory episodes, stereotypic counts, average velocity, and jump counts in the same locomotor activity setup. We investigated those parameters, as increased stereotypy in the open field reflects neuronal damage, brain lesions, or environmentally contributing factors (Fowler 2010), and possible changes in velocity reflect abnormal gait and other locomotor deficits originating from affected neuronal systems (Broom et al. 2017). Moreover, to identify whether treatment affects anxiety-related behaviours and thigmotaxis in the tested mice (Simon et al. 1994), the locomotion arena was divided to an “inner” zone (dimensions: startX = 2.5, startY = 2.5, endX = 14, endY = 14) and a residual “outer” zone closer to the walls of the arena. In this analysis, zone entries and the percentage of time spent in the “inner” zone to the total zone time were assessed. Indeed, increased thigmotaxis reflects anxiety-like responses in rodents, whereas decreased thigmotaxis is associated with increased impulsivity and possible cognitive deficits (Simon et al. 1994).

#### Repeated sCT pre-administration on the ability of acute alcohol to cause locomotor stimulation in mice

The mice were injected daily, at the same time every day, with sCT (5 μg/kg, IP; *N* = 16) or an equal amount of vehicle (saline solution, IP; *N* = 16) and always in the same room where the locomotor activity experiments were conducted. Following 48 h of no injections, the mice were tested in the locomotor activity boxes as described above. They were allowed to habituate in the testing boxes for an hour while baseline locomotion was recorded. They were subsequently injected with either alcohol (1.75 g/kg, IP) or an equal amount of vehicle (saline solution, IP), so that the following treatment groups were created: vehicle-vehicle, vehicle-alcohol, sCT-vehicle, and sCT-alcohol. Locomotor activity was registered for another 60 min, starting 5 min after the last vehicle or alcohol injection. Immediately after the termination of the test, the mice were decapitated and the brains were rapidly isolated and snap frozen in − 80 °C for further western blot analysis.

#### Repeated sCT and alcohol administration on alcohol-induced locomotor stimulation in mice

To further investigate the link between repeated sCT and alcohol-induced locomotion, we tested the effects of 5 daily administrations of sCT (5 μg/kg, IP) and alcohol (1.75 g/kg, IP) on alcohol-induced locomotor stimulation. The mice (*N* = 32) were subjected to 5 daily injections of sCT (5 μg/kg, IP) or an equal amount of vehicle (saline solution, IP) along with an alcohol (1.75 g/kg, IP) or an equal amount of vehicle (saline solution, IP) injection. Each day, the mice were allowed to habituate in the testing boxes for 30 min, while baseline locomotion was recorded. Subsequently, an sCT or vehicle injection 30 min prior to an alcohol or vehicle injection was administered, thus creating the following treatment groups: vehicle-vehicle, vehicle-alcohol, sCT-vehicle, and sCT-alcohol. Since the mice were given both injections daily, locomotor activity was then registered for another 30 min, starting 5 min after the last vehicle or alcohol injection.

### Tissue isolation

#### Western blot

The mice used at the repeated sCT-alcohol administration (experiment one) were decapitated immediately after the end of the experiment. The whole brain was rapidly isolated and placed into plastic tubes and snap frozen in − 80 °C. Further dissection of the LDTg, VTA, and the whole NAc region (both core and shell subregions) was performed by punching the aforementioned areas from frozen tissue. Specifically, brains were placed in a cold mouse brain matrix (Zivic instruments, Pittsburg, PA, USA) and coronally sectioned in 1-mm slices rostral to the fusion of the optic nerves with the optic chiasm according to the brain atlas (Paxinos and Franklin 2012). The desired section was placed under a stereoscope on a very cold glass plate (mix of dry ice and regular ice) to avoid tissue degradation, and the areas were isolated bilaterally, using a tissue biopsy punch (Zivic instruments, Pittsburg, PA, USA).

#### Ex vivo biochemical analysis

To further investigate our behavioural findings from the locomotor activity experiments following sCT treatment discontinuation, we measured monoamines and their metabolites in reward-related brain regions including VTA, the whole NAc region (both core and shell subregions), hippocampus, dorsal striatum, amygdala, and PFC. A separate group of mice (vehicle: *N* = 9, sCT: *N* = 8) were injected daily with sCT (5 μg/kg, IP) or an equal amount of vehicle (saline solution, IP) for 5 days. After 2 days without receiving injections, the mice were decapitated and brains were rapidly transferred on a cold glass plate for dissection. The brain regions were isolated and placed into plastic tubes, which were rapidly stored in − 80 °C until further analysis, as described previously (Prieto-Garcia et al. 2015).

### Western blot

For the western blot experiments, the isolated tissue was homogenised using a sonicator ultrasound homogenisation (Sonifier Cell Disruptor B30, Branson Sonic Power Co. Danbury, CT, USA) in a homogenisation buffer consisting of PBS, 0.1% Triton X-100, a protease-inhibitor cocktail tablet, and 5-mM EDTA. Following homogenisation, protein concentration determination was performed using the BCA (bicinchoninic acid) biochemical assay utilising a Quick Start Bovine Serum Albumin kit (Bio-Rad, Hercules, CA, USA). The amount of protein to be loaded on the gels was further calculated to 10 μg per sample. For the VTA and LDTg, the amount of protein calculated was that of 12 μg per sample, due to smaller amounts of tissue and protein present. The samples were mixed with a 2x Laemmli Sample Buffer (Bio-Rad, Hercules, CA, USA) containing β-mercaptoethanol and were loaded on a Mini-PROTEAN® TGX™ Precast Gel (Bio-Rad, Hercules, CA, USA). On all gels, a ScanLater Western Blot Protein Ladder™ was loaded as a marker for the identification of the molecular weight of the loaded proteins The electrophoresis took place in a Mini-PROTEAN® Tetra Vertical Electrophoresis Cell (Bio-Rad, Hercules, CA, USA), and the gel was run at 20 V for 5 min and at 300 V for another 18 min. The gel was then transferred on a PVDF membrane using the Trans-Blot® Turbo™ Mini PVDF Transfer Packs (Bio-Rad, Hercules, CA, USA), which was placed in a Trans-Blot® Turbo™ Transfer System (Bio-Rad, Hercules, CA, USA). The membrane was blocked in TBS-T (tris-buffered saline (custom made) plus Tween® 20 (Merck Millipore, Burlington, MA, USA)) containing 5% blotting-grade blocker nonfat milk powder (Bio-Rad, Hercules, CA, USA) for 1 h in room temperature. The membrane was incubated in primary antibody solutions overnight. After optimisation of the protocol, the final primary antibody solutions were as follows: the protein of interest anti-CTR (recognising two isoforms of the CTR, namely CTRa and CTRb; ID: *ab11042*, Abcam, Cambridge, UK) was diluted 1:1000 in TBS-T solution, and the reference protein anti-COX IV (ID: *ab14744*, Abcam, Cambridge, UK) was diluted 1:1000 in TBS-T + 5% nonfat dry milk solution. The membrane was incubated in a secondary antibody solution the next day for 1 h in room temperature. After protocol optimisation, the final secondary antibody solutions were as follows: 1:5000 in TBS-T of Eu-labelled goat anti-mouse ScanLater™ (Molecular Devices, San Jose, CA, USA) was used against a-COXIV, and 1:5000 in TBS-T of Eu-labelled goat anti-rabbit ScanLater™ (Molecular Devices, San Jose, CA, USA) was used against a-CTR. In between treatments, the membrane was subjected to various washing steps with TBS-T. The membrane was allowed to fully dry after incubation and was later visualised in a SpectraMax i3v Platform (Molecular Devices, San Jose, CA, USA).

### HPLC detection of monoamines and metabolites

Dissected brain tissue samples were homogenised by ultrasound homogenisation (Sonifier Cell Disruptor B30, Branson Sonic Power Co. Danbury, CT, USA) in a solution of 0.1-M perchloric acid, 5.37-mM EDTA, and 0.65-mM glutathione. After centrifugation (10,000 rpm, 5 **°**C, 10 min), the supernatant was collected and analysed for noradrenaline (NA); dopamine (DA); and its metabolites 3-methoxytyramine (3-MT), homovanillic acid (HVA), and 3,4-dihydroxyphenylacetic acid (DOPAC), as well as serotonin (5-HT) and its metabolite 5-hydroxyindoleacetic acid (5-HIAA), using a split fraction HPLC-ED system. The amines, dopamine, serotonin, noradrenaline, and 3-MT were analysed in an ion-exchange column (Nucleosil, 5 μ SA, 100 A, 150 × 2 mm; Phenomenex, Torrance, CA, USA) with a mobile phase consisting of 13.3-g citric acid, 5.84-g NaOH, 40-mg EDTA, and 200-ml methanol in distilled water to a total volume of 1000 ml. The acids, DOPAC, HVA, 5-HIAA were analysed in a reverse-phase column (Nucleosil, 3 μ, C18, 100 A, 50 × 2 mm, Phenomenex) with a mobile phase consisting of 11.22-g citric acid, 3.02-g dipotassium phosphate, 40-mg EDTA, and 60-ml methanol in distilled water to a total volume of 1000 ml. Electrochemical detection was performed by two amperometric detectors (Waters 460), and the currents were recorded with the Dionex Chromeleon software package (Dionex, Sunnyvale, CA, USA).

### Statistical analysis

Locomotor activity experiment one was analysed with two-way ANOVA for comparison of the total locomotor activity between treatments, and with two-way repeated measures ANOVA for comparison between treatment groups across time points of the recorded session. The secondary behavioural parameters measured in this locomotor activity test were analysed with two-way ANOVA. Locomotor activity experiment two was analysed using two-way repeated measures ANOVA for the comparison between treatments across time points. The secondary behavioural parameters measured in this locomotor activity test were analysed with repeated measures two-way ANOVA. All the aforementioned analyses were followed by using Tukey’s post hoc test for multiple comparisons. The ex vivo HPLC data were assessed with an unpaired two-tailed *t* test for the comparison between treatments. The western blot bands were quantified using the ImageJ software programme (public domain software; NIH, MD, USA), and the data were analysed using the non-parametric Kruskal-Wallis *H* test on the normalised intensity ratios (protein of interest (CTRa, CTRb)/protein of reference (COXIV)). One-way ANOVA analysis was performed on the raw intensity data of COXIV, to assess possible changes of the reference protein between treatment groups. Data are presented as mean ± SEM. The western blot data are presented as medians with interquartile range. A probability value of *P* < 0.05 was considered as statistically significant.

## Results

### Effects of repeated sCT pre-administration on the ability of acute alcohol to cause locomotor stimulation in mice

**Fig. 1:**
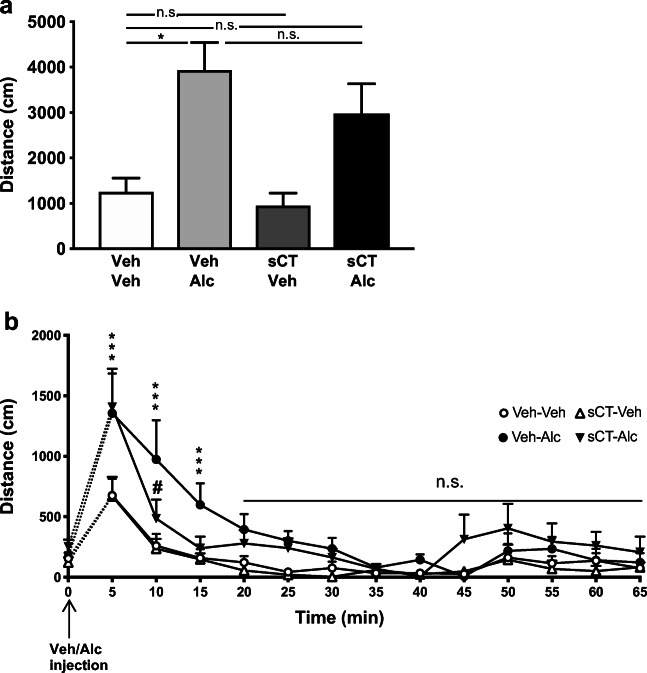
Acute alcohol does not induce locomotor stimulation in mice previously receiving 5-day sCT treatment when compared with the vehicle group. **a** Repeated sCT pre-administration (5 μg/kg, IP) for 5 days did not block locomotion caused by an acute alcohol injection (Alc, 1.75 g/kg, IP) administered on day 7 after sCT discontinuation. **b** Alcohol increased locomotion in mice at the time points of 5, 10, and 15 min after administration. After treatment discontinuation, sCT sustained an inhibitory effect on this alcohol-induced locomotion at the time point of 10 min after alcohol injection in the same behavioural setup. (Data are presented as mean ± SEM; *P < 0.05, ***P < 0.001 for Veh-Alc vs Veh-Veh, ^#^*P* < 0.05 for sCT-Alc vs Veh-Alc comparisons; n.s., non-significant)

The analysis of the effect of sCT pre-treatment for 5 days and acute alcohol on day 7 on the cumulative 60-min locomotor activity in mice (*N* = 8 per group) showed an overall effect of alcohol treatment (F(1, 28) = 16.08, *P* = 0.0004; Fig. 1a). However, no significant effect of sCT treatment or alcohol × sCT treatment interaction was observed. Further analysis revealed that alcohol increased locomotor stimulation in mice pre-treated with vehicle compared to the vehicle-vehicle group (*P* < 0.05). Alcohol treatment did not induce locomotor stimulation in mice pre-treated with sCT compared with vehicle; however, no significant effect was noted between the vehicle-alcohol and sCT-alcohol groups.

The analysis of locomotor activity over time in the same mice showed an overall effect of treatment (F(3, 28) = 6.72, *P* = 0.0015; Fig. 1b), similar to time (F(12, 336) = 21.23, *P* < 0.0001) and time × treatment interaction (F(36, 336) = 1.53, *P* = 0.0297). Alcohol significantly increased locomotor activity in animals pre-treated with vehicle compared with the vehicle (*P* < 0.001) group at 5 min after the alcohol challenge. At the same time point, alcohol significantly enhanced locomotion in the group previously treated with sCT when compared with the vehicle (*P* < 0.0001) group. There was no difference between the vehicle and sCT-vehicle groups or between the alcohol and sCT-alcohol groups. At 10 min after alcohol administration, the vehicle-alcohol group showed increased locomotion compared with the vehicle (*P* < 0.0001)-pre-treated group. At the same time point, alcohol-induced locomotor stimulation in the sCT-pre-treated group was significantly lower (*P* < 0.05) compared with the vehicle-alcohol group. There were no differences between the vehicle and sCT-vehicle or sCT-alcohol groups. Alcohol increased locomotor activity at the 15-min time point after administration to the vehicle (*P* < 0.05)-pre-treated group when compared with vehicle. There were no other differences noted between treatment groups at any other time point.

### Effects of repeated sCT pre-administration and acute alcohol challenge on secondary behavioural parameters in mice

Supplementary Fig. 1 (Online Resource 1) shows the detailed analysis of the secondary behavioural parameters measured in the previously described locomotor activity experiment in male mice.

### Effects of repeated sCT and acute alcohol challenge on the levels of the CTR a and b in mice brain areas

In the same group of mice as in locomotor activity experiment one, there was no effect of treatment on the levels of the reference protein COXIV in the LDTg, VTA, or NAc (Online Resource 2).

In the LDTg (Supplementary Fig. 2a), no effect of treatment was noted on the CTRa levels (mean ranks for vehicle-vehicle = 10.29, *N* = 7; vehicle-alcohol = 16.00, *N* = 7; sCT-vehicle = 14.33, *N* = 6; and sCT-alcohol = 13.50, *N* = 6; Supplementary Fig. 2b) normalised to COXIV. Due to low-intensity bands for CTRb in the LDTg area, further statistical analysis was not performed (*N* < 3).

In the VTA (Supplementary Fig. 2c), no effect of treatment was found for the normalised CTRa (mean ranks for vehicle-vehicle = 13.25, *N* = 8; vehicle-alcohol = 15.13, *N* = 8; sCT-vehicle = 19.71, *N* = 7; and sCT-alcohol = 16.38, *N* = 8; Supplementary Fig. 2d) and CTRb levels (mean ranks for vehicle-vehicle = 11.14, *N* = 7; vehicle-alcohol = 12.57, *N* = 7; sCT-vehicle = 13.40, *N* = 5; and sCT-alcohol = 13.40, *N* = 5; Supplementary Fig. 2e).

Analysis of the NAc bands (Supplementary Fig. 2f) did not show any statistical differences in the protein levels of either isoform CTRa (mean ranks for vehicle-vehicle = 16.50, *N* = 8; vehicle-alcohol = 13.50, *N* = 8; sCT-vehicle = 20.17, *N* = 6; and sCT-alcohol = 10.57, *N* = 7; Supplementary Fig. 2 g) or CTRb levels (mean ranks for vehicle-vehicle = 15.13, *N* = 8; vehicle-alcohol = 16.38, *N* = 8; sCT-vehicle = 17.67, *N* = 6; and sCT-alcohol = 11.00, *N* = 7; Supplementary Fig. 2 h), as normalised to COXIV. Supplementary Fig. 3 (Online Resource 3) shows a representative visualised western blot membrane for each studied area.

### Effects of repeated sCT administration on monoamines and their metabolites in mice brain areas

**Fig. 2:**
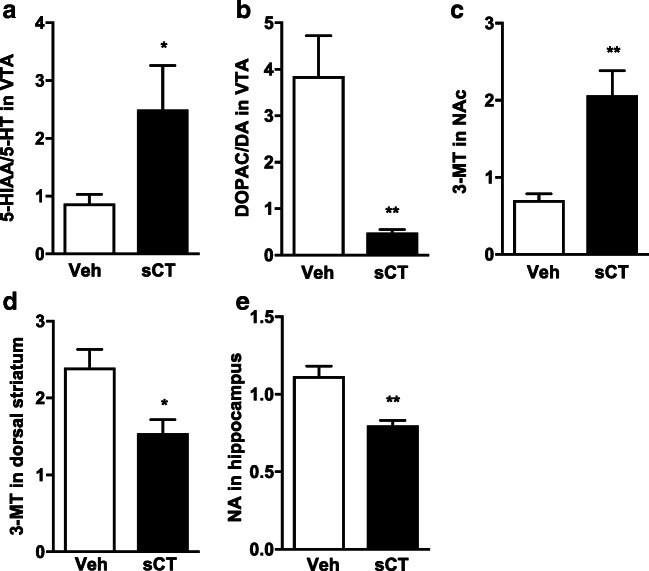
Sub-chronic sCT administration alters monoamines and their metabolite levels in mice brain areas. Repeated sCT administration (5 μg/kg, IP) for 5 days increased the levels of the **a** 5-HIAA/5-HT and decreased the **b** DOPAC/DA turnover in the VTA of mice when compared with vehicle (Veh). sCT increased the levels of **c** 3-MT in the NAc, decreased **d** 3-MT in the dorsal striatum of mice, and decreased **e** NA in the hippocampus. (Data are presented as mean ± SEM; *P < 0.05, **P < 0.01)

The ex vivo analysis revealed that repeated sCT administration for 5 days significantly increased the 5-HIAA/5-HT (*P* = 0.0441, vehicle: *N* = 9, sCT: *N* = 8; Fig. 2a) turnover, whereas it decreased the DOPAC/DA (*P* = 0.0025, Fig. 2b) turnover in the VTA compared with vehicle-pre-treated mice. Moreover, sCT increased concentration of 3-MT (*P* = 0.0015, vehicle: *N* = 9, sCT: *N* = 8; Fig. 2c) in the NAc and decreased 3-MT in the dorsal striatum (*P* = 0.0107, vehicle: *N* = 8, sCT: *N* = 9; Fig. 2d) compared with the vehicle treatment in mice. Lastly, sCT decreased NA levels (*P* = 0.0014, vehicle: *N* = 9, sCT: *N* = 7; Fig. 2e) in the hippocampus of mice. No other significant effects were observed for other monoamines or metabolites in the investigated areas as shown in the Supplementary Table 1 (Online Resource 4).

### Effects of repeated sCT and alcohol administration on alcohol-induced locomotor stimulation in mice

**Fig. 3:**
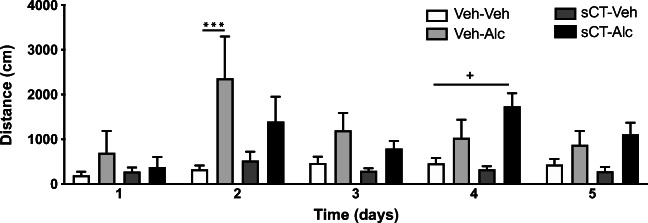
Sub-chronic sCT and alcohol administration differentially affects alcohol-induced locomotor stimulation in mice. The noted increase in locomotor response to alcohol (Alc, 1.75 g/kg, IP) is not evident during the first 3 days in mice pre-treated with sCT (5 μg/kg, IP) for 5 days. sCT enhances the locomotor stimulation caused by alcohol on experimental day 4. (Data are presented as mean ± SEM, ***P < 0.001 for Veh-Alc vs Veh-Veh, ^+^*P* < 0.05 for sCT-Alc vs Veh-Veh; n.s., non-significant)

The analysis of locomotor stimulation in mice (*N* = 8 per group) after 5 days of sCT and alcohol administration showed an overall effect of treatment (F(3, 28) = 6.61, *P* = 0.0016; Fig. 3) and time (F(4, 112) = 3.34, *P* = 0.0127), but not of time × treatment interaction. No differences were noted in locomotor activity between the treatment groups on day 1 of the experiment. On day 2, alcohol significantly increased locomotor activity in comparison with the vehicle (*P* < 0.001) group. On the same day, sCT decreased but did not block alcohol-induced locomotor stimulation when compared with the vehicle-alcohol group. There were no differences in locomotor stimulation between the treatment groups on day 3. Interestingly, sCT administration increased alcohol-induced locomotor stimulation on day 4 when compared with the vehicle (*P* < 0.05) group, but no difference was noted when compared with the alcohol group. No differences were found between treatments on day 5.

### Effects of repeated sCT and alcohol administration on secondary behavioural parameters of the locomotor activity experiment in male mice

Supplementary Fig. 2 (Online resource 5) shows the detailed analysis of the secondary behavioural parameters measured in the previously described locomotor activity experiment in male mice

## Discussion

Here, we present additional data to the established inhibitory effects of sCT on alcohol-mediated behaviours (Kalafateli et al. 2019a; Kalafateli et al. 2019b) as we attempt to identify the less studied locomotor stimulatory and molecular effects of repeated sCT-alcohol administration. We firstly show that acute alcohol does not induce locomotion in mice pre-treated with sCT when they are compared with the vehicle group, and this effect is not accompanied by the difference in the protein levels of the CTR a and b in reward-related areas of the same mice. Secondly, our ex vivo biochemical data indicate that these behavioural effects might be related to altered monoamines and their metabolites in the investigated reward-related brain areas in mice. Lastly, we found a differential effect of sCT on alcohol-induced locomotor stimulation when sCT is administered with alcohol for 5 days.

We here demonstrate that after 5 days of sCT administration, an acute alcohol challenge did not increase locomotion in the previously sCT-treated mice compared with the vehicle group. Although there were no significant differences between the vehicle and sCT-pre-treated mice after an alcohol injection, our data are in accordance with previous studies on ghrelin, another food intake-regulating peptide, showing that sub-chronic pre-administration of a ghrelin receptor antagonist decreases the acute alcohol-evoked locomotion in mice without the antagonist being present (Suchankova et al. 2015). As sCT is no longer present in our tests, a possible long-term modulation of areas important for reward by this agent might be suggested. However, this is not linked to changes in the levels of CTR, as in mice of the same experiments, we did not detect any differences between treatments in the CTR levels in the LDTg, VTA, or NAc, areas important in the expression of alcohol locomotor stimulatory properties (Blomqvist et al. 1997; Gatto et al. 1994; Larsson et al. 2005; Tupala and Tiihonen 2004). This lack of effect in receptor levels is in line with results that repeated ghrelin receptor antagonist decreases alcohol-induced locomotion after discontinuation without detected differences in the expression of ghrelin receptors (Suchankova et al. 2015). In further accordance with our data, amylin administration does not affect the mRNA expression levels of the CTRa in rats but alters the mRNA levels of RAMPs 1–3 (Liberini et al. 2016). Therefore, the locomotor differences noted in the present locomotion experiments could be associated with altered RAMP levels; the lack of data thereof can be considered as a limitation, and future studies are warranted. Although differential expression of the calcitonin receptor gene is detected in the NAc of high compared with low alcohol-consuming rats chronically exposed to alcohol (Kalafateli et al. 2019a), the lack of effect on the protein levels of this receptor in the present could be a result of chronic alcohol exposure in rats, which is not the case in the present conditions in mice. Additionally, previous studies have showed differences in amylin-mediated behaviours between the subregions of the NAc (Baldo and Kelley 2001; Mietlicki-Baase et al. 2015b). Since in the current studies the whole NAc region was isolated, possible evaluation of each subregion might reveal differential levels of the CTR. Although we did not detect any treatment effects in this study, our data confirm previous studies establishing that mRNA of the calcitonin receptor gene is present in all the three aforementioned areas in rats (Mietlicki-Baase et al. 2017; Reiner et al. 2017; Sexton et al. 1994). Moreover, we found that CTRa is the most abundant isoform of the receptor in all areas studied herein, which is in accordance with rat studies showing a 5-fold higher expression of the CTRa over CTRb gene in the LDTg (Reiner et al. 2017) and the sole expression of the CTRa isoform in area postrema (Liberini et al. 2016).

It should also be noted that the additional behavioural analysis of this locomotor activity experiment did not show a significant effect of sCT pre-treatment on any of the measured secondary behavioural parameters. These results indicate that sCT affects the ability of alcohol to activate the mesolimbic dopamine system, as expressed by increased locomotion (Phillips and Shen 1996), without influencing behaviours such as gait, impulsivity, anxiety-like behaviours, or neuronal dysfunction (Broom et al. 2017; Fowler 2010; Simon et al. 1994).

Our ex vivo biochemical data may provide another tentative explanation for the ability of repeated and terminated sCT to reduce the behavioural outcome of alcohol. In the VTA, sCT administration for 5 days increases the 5-HIAA/5-HT ratio and decreases the DOPAC/DA ratio, possibly suggesting that these mechanisms inhibit alcohol from activating the dopamine projections in this area (Gessa et al. 1985). Consistent with the present biochemical effect, amylin injected into the hypothalamus increases the brain metabolism of serotonin and dopamine in various rat brain areas (Chance et al. 1992). The present VTA-dopamine biochemical data could be explained by the presence of amylin receptors on dopaminergic neurons in the VTA (Mietlicki-Baase et al. 2015b) and by the fact that sCT into the VTA reduces food reward-induced dopamine release in the rat NAc (Mietlicki-Baase et al. 2015b). The additional findings that sCT sub-chronic administration increases the levels of 3-MT in the NAc and decreases them in the dorsal striatum and reduces noradrenaline levels in the hippocampus of mice, areas of importance for alcohol behavioural responses (Di Chiara and Imperato 1986; Robbins and Everitt 2002; Sun et al. 2018), could also contribute to the attenuated locomotor response to alcohol noted in our behavioural experiments. However, biochemical experiments in other brain areas, for example, those that are involved in motor functions, are warranted in the future.

In contrast to an attenuation of alcohol-induced locomotor stimulation as previously observed (Kalafateli et al. 2019b), we here notice that the alcohol response is greater in sCT-treated mice compared with that in vehicle-treated mice. However, co-administration of sCT and alcohol for 5 days did not alter any secondary behavioural parameters as measured in this locomotor activity experiments. Collectively, this indicates that the behavioural response to sCT in combination with alcohol changes over exposure time. This observation might explain our previous results showing that 3-day administration of sCT initially reduces alcohol intake in rats with a return to baseline alcohol intake on the last day of administration (Kalafateli et al. 2019a). Chronic sCT infusions show similar tolerance patterns for food intake, which suggestively develop after 3 days (Chelikani et al. 2007), raising points of consideration for the use of sCT in the clinical setting. sCT has been shown to act as a selective amylin receptor agonist, but it also activates the CTR per se without the presence of RAMPs (Lee et al. 2016); therefore, further studies with more selective and potent amylin receptor agonists would be of great interest.

The present findings add novelty to the alcohol-amylin field by demonstrating that sCT affects alcohol’s ability to activate the mesolimbic dopamine system, as expressed by enhanced locomotion, without influencing secondary behavioural parameters. This appears to involve altered monoaminergic tone in certain brain areas in mice, rather than affecting the levels of central *CTR* per se. Despite the need for further translatable experiments, the present studies offer a more mechanistic perspective on how sCT influences alcohol-induced locomotor stimulation. Further illumination of the properties and the mechanisms of action of sCT can provide better understanding of the ability of this agent to regulate alcohol behaviours.

## Electronic supplementary material

ESM 1 (PDF 123 kb)

ESM 2 (PDF 133 kb)

ESM 3 (PDF 229 kb)

ESM 4 (DOCX 21 kb)

ESM 5 (DOCX 21 kb)
