# Supplementary material for: Effects of sub-chronic amylin receptor activation on alcohol-induced locomotor stimulation and monoamine levels in mice
Source: Psychopharmacology (Berl). 2020 Jul 10;237(11):3249–57. doi: 10.1007/s00213-020-05607-8 (PMC7561575; doi:10.1007/s00213-020-05607-8)
Supplement: Supplementary file 1 — (PDF 123 kb) [file 213_2020_5607_MOESM1_ESM.pdf]

Supplementary Figure 1

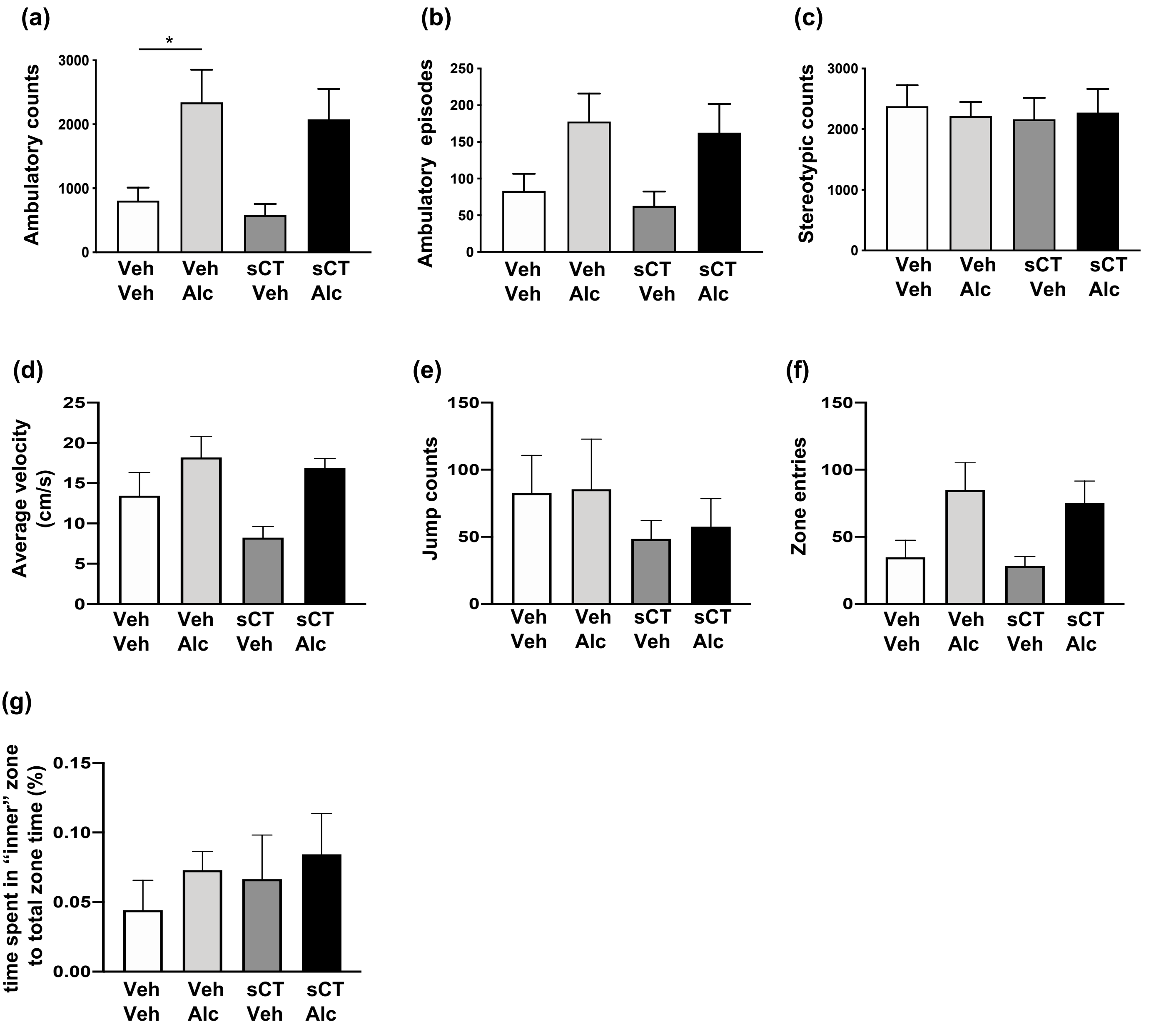

Supplementary Figure 1. Effects of repeated sCT pre-treatment and acute alcohol challenge on secondary behavioural parameters in the locomotor activity experiment in male mice.

(a) There was an overall effect of alcohol treatment ($F(1, 28)=16.40$, $P=0.0004$), but not of sCT pre-treatment $F(1, 28)=0.43$, $P=0.5184$) or treatment interaction ($F(1, 28)=0.003$, $P=0.957$) on ambulatory counts. Post hoc analysis showed that alcohol increased ambulatory counts in mice pre-treated with vehicle when compared to the vehicle group ($P=0.0340$). **(b)** An overall effect of alcohol $F(1, 28)=9.66$ $P=0.0043$), but not of pre-treatment ($F(1, 28)=0.33$ $P=0.5708$), or treatment interaction ($F(1, 28)=0.007$ $P=0.9353$**)** was noted on ambulatory episodes. **(c)** No effect of pre-treatment ($F(1, 28)=0.06$ $P=0.8121$), alcohol ($F(1, 28)=0.005$, $P=0.9445$), or their interaction ($F(1, 28)=0.16$,$P=0.6912$) was noted on stereotypic counts. **(d)** There was an overall effect of alcohol ($F(1, 28)=9.65$, $P=0.0043$), but not of sCT pre-treatment ($F(1, 28)=2.28$, $P=0.1424$) or their interaction ($F(1, 28)=0.82$, $P=0.3722$) on average velocity. **(e)** No effect of pre-treatment ($F(1, 28)=1.36$ $P=0.2528$), alcohol ($F(1, 28)=0.05$, $P=0.8250$) or their interaction ($F(1, 28)=0.01$, $P=0.9092$) was noted on jump counts. **(f)** There was an overall effect of alcohol ($F(1, 28)=10.55$, $P=0.0030$), but not of sCT ($F(1, 28)=0.29$, $P=0.5940$) or treatment interaction ($F(1, 28)=0.01$, $P=0.9109$) on zone entries. Lastly, **(g)** sCT pre-treatment ($F(1, 28)=0.45$, $P=0.5074$), alcohol ($F(1, 28)=0.86$, $P=0.3623$) or treatment interaction ($F(1, 28)=0.05$, $P=0.8303$) had no effect on the time the mice spent in the "inner" zone to the total zone time in the arena.
Two-way ANOVA analysis with results shown as F(DFn, DFd), $*P<0.05$, data are represented in the graphs as mean$\pm$SEM.
