# Supplementary material for: Effects of sub-chronic amylin receptor activation on alcohol-induced locomotor stimulation and monoamine levels in mice
Source: Psychopharmacology (Berl). 2020 Jul 10;237(11):3249–57. doi: 10.1007/s00213-020-05607-8 (PMC7561575; doi:10.1007/s00213-020-05607-8)
Supplement: Supplementary file 2 — (PDF 133 kb) [file 213_2020_5607_MOESM2_ESM.pdf]

Supplementary Figure 2

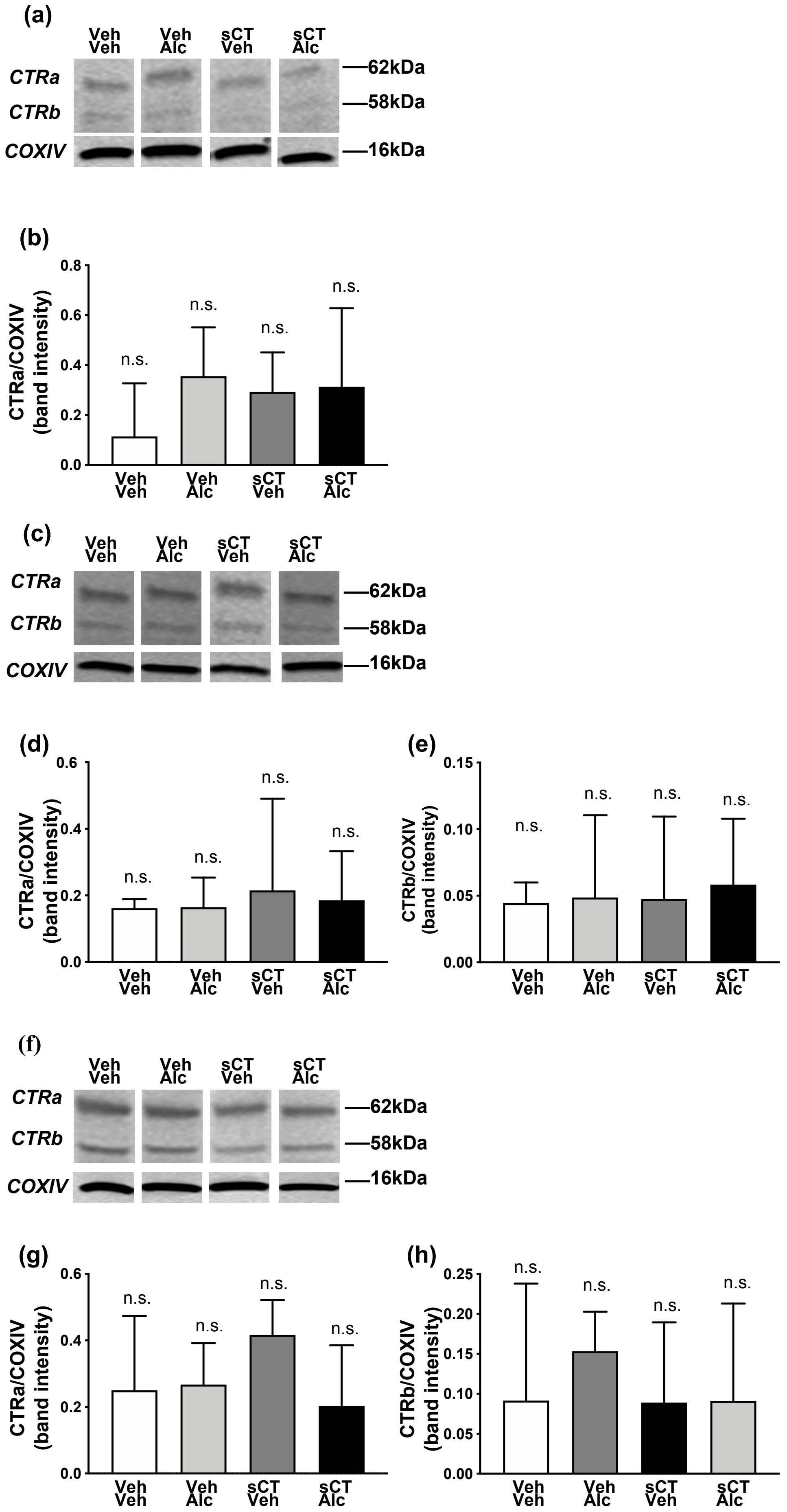

Supplementary Figure 2. Discontinuation of sub-chronic sCT pre-administration and acute alcohol administration do not affect CTR levels in reward-related areas in mice

Repeated sCT pre-administration (5 µg/kg, IP) for 5 days and an alcohol (1.75 g/kg, IP) injection on day 7 after sCT discontinuation, does not affect the levels of **(a, b)** CTRa in the LDTg, **(c, d)** CTRa and **(c, e)** CTRb in the VTA and **(f, g)** CTRa and b in the NAc **(f, h)** in the mice from the behavioural locomotion experiments. (Data are presented as mean ±SEM of relative intensity, n.s.: non-significant; Veh: vehicle, Alc: alcohol)
