# Supplementary material for: Effects of sub-chronic amylin receptor activation on alcohol-induced locomotor stimulation and monoamine levels in mice
Source: Psychopharmacology (Berl). 2020 Jul 10;237(11):3249–57. doi: 10.1007/s00213-020-05607-8 (PMC7561575; doi:10.1007/s00213-020-05607-8)
Supplement: Supplementary file 3 — (PDF 229 kb) [file 213_2020_5607_MOESM3_ESM.pdf]

Supplementary Figure 3

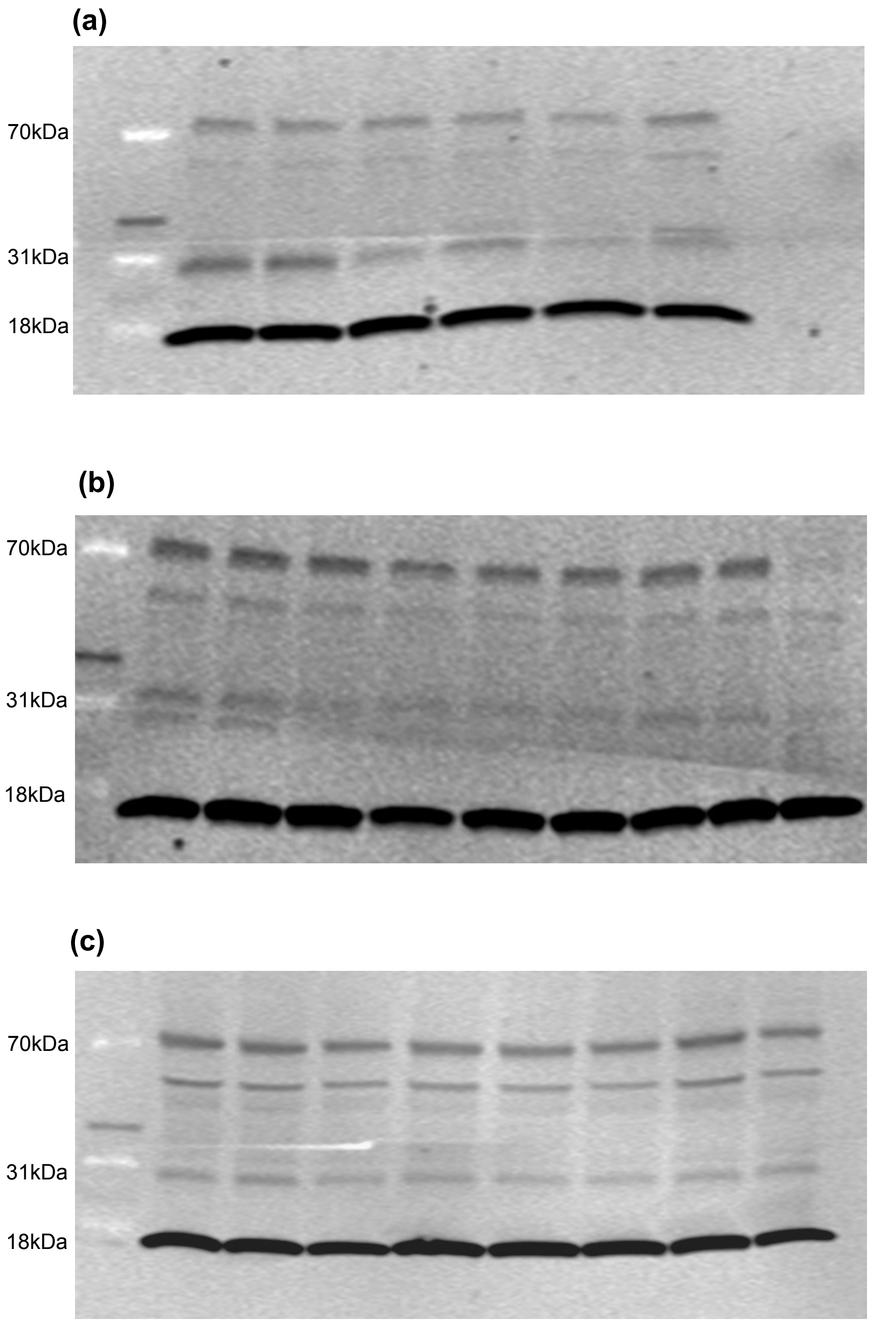

Supplementary Figure 3. Representative membranes from the Western Blot experiments

Visualised Western Blot gel membranes for **(a)** LDTg, **(b)** VTA and **(c)** NAc.
