# Supplementary material for: Effects of sub-chronic amylin receptor activation on alcohol-induced locomotor stimulation and monoamine levels in mice
Source: Psychopharmacology (Berl). 2020 Jul 10;237(11):3249–57. doi: 10.1007/s00213-020-05607-8 (PMC7561575; doi:10.1007/s00213-020-05607-8)
Supplement: Supplementary file 4 — (DOCX 21 kb) [file 213_2020_5607_MOESM4_ESM.docx]

|  | | **NA** | **DA** | **DOPAC** | **HVA** | **DOPAC/DA** | **HVA/DA** | **(DOPAC+HVA)/DA** | **3-MT** | **5-HT** | **5-HIAA** | **5-HIAA/**  **5-HT** |
| --- | --- | --- | --- | --- | --- | --- | --- | --- | --- | --- | --- | --- |
| **VTA** | ***Veh*** | 1.7 ± 0.2 n=9 | 0.6 ± 0.2  n=9 | 1.3 ± 0.2 n=9 | 2.1 ± 0.4  n=9 | - | 6.3 ± 1.5 n=9 | 10.2 ± 2.4  n=9 | - | 2.1 ± 0.3 n=9 | 1.5 ± 0.2  n=9 | - |
|  | ***sCT*** | 1.8 ± 0.2 n=8 | 0.8 ± 0.2 n=8 | 1.5 ± 0.2 n=8 | 2.1 ± 0.2  n=7 | - | 3.5 ± 0.8 n=7 | 6.2 ± 1.5  n=7 | - | 2.4 ± 0.2 n=8 | 1.3 ± 0.1 n=8 | - |
|  | ***P value*** | 0.9451 | 0.6520 | 0.5963 | 0.8644 | - | 0.0843 | 0.2081 | - | 0.6339 | 0.4603 | - |
| **NAc** | ***Veh*** | 1.8 ± 0.2 n=9 | 20.2 ± 2.0 n=9 | 2.3 ± 0.1 n=9 | 1.9 ± 0  n=9 | 0.1 ± 0  n=9 | - 1. ± 0   n=9 | - 1. ± 0   n=9 | 2.9 ± 0.1  n=9 | 8.2 ± 0.6  n=9 | 2.9 ± 0.2 n=9 | 1.8 ± 0.2  n=9 |
|  | ***sCT*** | 2.2 ± 0.2 n=9 | 21.9 ± 3.0 n=9 | 2.8 ± 0.4 n=9 | 2.2 ± 0.4  n=9 | 0.1 ± 0  n=9 | - 1. ± 0   n=9 | - 1. ± 0   n=9 | 3.3 ± 0.4  n=9 | 7.4 ± 0.9  n=9 | 2.3 ± 0.2 n=9 | 2.2 ± 0.2  n=9 |
|  | ***P value*** | 0.2371 | 0.6389 | 0.2401 | 0.4027 | 0.2641 | 0.3559 | 0.5204 | 0.2942 | 0.4729 | 0.0885 | 0.2371 |
| **Hippo-**  **campus** | ***Veh*** | 0 ± 0  n=9 | 0.8 ± 0.1  n=9 | 1.2 ± 0.2 n=9 | 17.8 ± 2.7  n=9 | 27.8 ± 3.7  n=9 | 45.6 ± 6.1 n=9 | **-** | 1.3 ± 0.2  n=9 | 0.1 ± 0 n=9 | 0.1 ± 0 n=9 | 0 ± 0  n=9 |
|  | ***sCT*** | 0 ± 0  n=7 | 0.5 ± 0  n=7 | 0.8 ± 0  n=7 | 16 ± 3.8  n=7 | 23.5 ± 5  n=7 | 39.5 ± 8.3 n=7 | **-** | 0.9 ± 0.1  n=7 | 0.2 ± 0 n=7 | 0.4 ± 0.2 n=7 | 0 ± 0  n=7 |
|  | ***P value*** | 0.8987 | 0.2023 | 0.0913 | 0.6989 | 0.2462 | 0.5530 | **-** | 0.1707 | 0.5281 | 0.3037 | 0.8987 |
| **Dorsal**  **Striatum** | ***Veh*** | 0.5 ± 0  n=8 | 18.2 ± 2.8 n=8 | 0.8 ± 0  n=8 | 1.3 ± 0.6  n=8 | 0 ± 0  n=8 | 0 ± 0  n=8 | - 1. ± 0   n=8 | 0.7 ± 0  n=8 | 5.2 ± 1.2  n=8 | 7.5 ± 1.6 n=8 | 0.5 ± 0  n=8 |
|  | ***sCT*** | 0.5 ± 0  n=9 | 19.1 ± 2.0 n=9 | 1.0 ± 0.1 n=9 | 0.8 ± 0.1  n=9 | 0 ± 0  n=9 | 0 ± 0  n=9 | 0 ± 0  n=9 | 0.9 ± 0.1  n=9 | 3.7 ± 0.5  n=9 | 4.3 ± 0.4 n=9 | 0.5 ± 0  n=9 |
|  | ***P value*** | 0.7041 | 0.7880 | 0.1561 | 0.3999 | 0.5748 | 0.1248 | 0.3218 | 0.1703 | 0.2821 | 0.0608 | 0.7041 |
| **Amygdala** | ***Veh*** | 5.6 ± 0.3 n=9 | 2.0 ± 0.3  n=9 | 2.6 ± 0.2 n=9 | 2.5 ± 0.1  n=9 | 1.4 ± 0.4  n=9 | 1.4 ± 0.1 n=9 | 2.8 ± 0.2  n=9 | 0 ± 0  n=9 | 3.7 ± 0.3  n=9 | 0.7 ± 0.1 n=9 | 0.2 ± 0  n=9 |
|  | ***sCT*** | 6.0 ± 0.3 n=8 | 1.7 ± 0  n=8 | 3.0 ± 0.2 n=8 | 2.3 ± 0.2  n=8 | 1.7 ±0.2  n=9 | 1.3 ± 0  n=8 | 3.0 ± 0.1  n=8 | 0 ± 0  n=8 | 4.1 ± 0.4 n=8 | 0.6 ± 0 n=8 | 0.2 ± 0  n=8 |
|  | ***P value*** | 0.4246 | 0.3817 | 0.2919 | 0.3156 | 0.0677 | 0.4396 | 0.2935 | 0.5257 | 0.4645 | 0.3258 | 0.1861 |
| **Prefrontal**  **Cortex** | ***Veh*** | 0.7 ± 0 n=9 | 0.2 ± 0  n=9 | 0.9 ± 0.1 n=9 | 1.1 ± 0.1  n=9 | 7.0 ± 1.6  n=9 | 8.2 ± 1.7 n=9 | 15.2 ± 3.2  n=9 | 0 ± 0  n=9 | 1.1 ± 0.1 n=9 | 0.5 ± 0.1 n=9 | 0.5 ± 0.2  n=9 |
|  | ***sCT*** | 0.7 ± 0 n=8 | 0.2 ± 0  n=8 | 0.8 ± 0.1 n=8 | 1.2 ± 0.1  n=8 | 6.0 ± 1.1  n=8 | 9.2 ± 2.5 n=8 | 15.2 ± 3.5  n=8 | 0 ± 0  n=8 | 1.2 ± 0.1 n=8 | 0.4 ± 0.1 n=8 | 0.4 ± 0.1  n=8 |
|  | ***P value*** | 0.8816 | 0.7455 | 0.9086 | 0.7436 | 0.6129 | 0.3645 | 0.9999 | 0.2699 | 0.8663 | 0.5364 | 0.6970 |
| Values are represented as mean ± SEM after unpaired ttest (significant level P<0.05). **NA**: noradrenaline, **DA**: dopamine, **5-HT**: serotonin | | | | | | | | | | | | |

**Supplementary Table 1.** Effects of sub-chronic five-day administration of sCT on monoamines and their metabolites in brain areas of male mice.
