# Supplementary material for: Effects of sub-chronic amylin receptor activation on alcohol-induced locomotor stimulation and monoamine levels in mice
Source: Psychopharmacology (Berl). 2020 Jul 10;237(11):3249–57. doi: 10.1007/s00213-020-05607-8 (PMC7561575; doi:10.1007/s00213-020-05607-8)
Supplement: Supplementary file 5 — (DOCX 21 kb) [file 213_2020_5607_MOESM5_ESM.docx]

|  | **Treatment** | **Time** | **Interaction** |
| --- | --- | --- | --- |
| **Ambulatory counts** | F (3, 28) = 0.15  P=0.9301 | F (4, 112) = 2.12  P=0.0819 | F (12, 112) = 1.09  P=0.3704 |
| **Ambulatory episodes** | F (12, 112) = 0.95  P=0.5000 | F (4, 112) = 1.83  P=0.1264 | F (12, 112) = 0.95  P=0.5000 |
| **Stereotypic counts** | F (3, 28) = 2.46  P=0.0832 | F (4, 112) = 0.14  P=0.9670 | F (12, 112) = 1.40  P=0.1739 |
| **Average velocity** | F (3, 28) = 1.63  P=0.2044 | F (4, 112) = 0.55  P=0.6959 | F (12, 112) = 1.02  P=0.4364 |
| **Jump counts** | F (3, 28) = 1.68  P=0.1938 | F (4, 112) = 1.75  P=0.1429 | F (12, 112) = 0.57  P=0.8545 |
| **Zone entries** | F (3, 28) = 0.09  P=0.9631 | F (4, 112) = 4.66  **P=0.0016** | F (12, 112) = 0.97  P=0.4753 |
| **% time spent in “inner” zone to total zone time** | F (3, 28) = 0.25  P=0.8605 | F (4, 112) = 4,50  **P=0.0021** | F (12, 112) = 0.71  P=0.7376 |
| Data shown as F (DFn, DFd) after two-way repeated measures ANOVA analysis; significance level of P<0.05. | | | |

**Supplementary Table 2.** Effects of repeated sCT and alcohol administration on secondary behavioural parameters in the locomotor activity experiment in male mice.
